# RNA-Seq analysis implicates dysregulation of the immune system in schizophrenia

**DOI:** 10.1186/1471-2164-13-S8-S2

**Published:** 2012-12-17

**Authors:** Junzhe Xu, Jingchun Sun, Jingchun Chen, Lily Wang, Anna Li, Matthew Helm, Steven L Dubovsky, Silviu-Alin Bacanu, Zhongming Zhao, Xiangning Chen

**Affiliations:** 1 Department of psychiatry, School of Medicine, University at Buffalo, SUNY, Buffalo, NY 14260, USA; 2 VA Western New York HealthCare System, Buffalo, NY 14215, USA; 3 Buffalo Psychiatric Center, Buffalo, NY 14213, USA; 4 Department of Biomedical Informatics, Vanderbilt University School of Medicine, Nashville, TN 37232, USA; 5 Department of Psychiatry, Vanderbilt University School of Medicine, Nashville, TN 37212, USA; 6 Virginia Institute for Psychiatric and Behavioral Genetics, Virginia Commonwealth University, Richmond, VA 23298, USA; 7 Department of Biostatistics, Vanderbilt University School of Medicine, Nashville, TN 37232, USA; 8 Department of Cancer Biology, Vanderbilt University Medical Center, Nashville, TN 37232, USA; 9 Department of Human and Molecular Genetics, Virginia Commonwealth University, Richmond, VA 23298, USA

## Abstract

**Background:**

While genome-wide association studies identified some promising candidates for schizophrenia, the majority of risk genes remained unknown. We were interested in testing whether integration gene expression and other functional information could facilitate the identification of susceptibility genes and related biological pathways.

**Results:**

We conducted high throughput sequencing analyses to evaluate mRNA expression in blood samples isolated from 3 schizophrenia patients and 3 healthy controls. We also conducted pooled sequencing of 10 schizophrenic patients and matched controls. Differentially expressed genes were identified by t-test. In the individually sequenced dataset, we identified 198 genes differentially expressed between cases and controls, of them 19 had been verified by the pooled sequencing dataset and 21 reached nominal significance in gene-based association analyses of a genome wide association dataset. Pathway analysis of these differentially expressed genes revealed that they were highly enriched in the immune related pathways. Two genes, *S100A8 *and *TYROBP*, had consistent changes in expression in both individual and pooled sequencing datasets and were nominally significant in gene-based association analysis.

**Conclusions:**

Integration of gene expression and pathway analyses with genome-wide association may be an efficient approach to identify risk genes for schizophrenia.

## Background

Schizophrenia is characterized by delusions, hallucinations, and deficits in cognitive function. Over the years, epidemiologic studies have accumulated significant evidence that many genetic factors play important roles in both symptomatology and etiology. Recent genetic studies, including genome-wide association (GWA) studies, have identified several promising candidate genes and loci. One of the most consistent findings in GWA studies is the major histocompatibility (MHC) region in 6p [1-3]. This finding strongly implicates the immune system as being involved in the development of schizophrenia. Other findings include candidate genes functioning in cell adhesion [4-6], migration [4,7] and apoptosis [8,9]. Except the findings from rare copy number variations [10-13], the effects of individual genes overall are modest or weak. These results suggest that many genes with moderate or small effects may be involved in schizophrenia [14]. It is difficult to identify these genes without increased sample sizes and better analysis of phenotype. Integration of other functional studies or utilization of high throughput technologies may increase the power to detect other schizophrenia candidate genes.

Whole genome mRNA sequencing (RNA-Seq, or transcriptome sequencing) allows for the comprehensive survey of all the mRNAs in a sample. This platform is the fruit of recently developed high-throughput DNA sequencing technology [15,16], and has produced exciting results in the study of various diseases [17-19]. In this experiment, we applied the RNA-Seq technology to study schizophrenia. Specifically, we aimed to understand the dysregulation in schizophrenia at higher levels of biological structure and to integrate gene expression data to facilitate the identification of promising candidate genes. To accomplish these goals, we sequenced the blood mRNAs isolated from 3 schizophrenic patients and 3 matched healthy controls. We verified the differentially expressed genes (DEGs) in 2 independent pooled samples. We further examined the association of the discovered DEGs using GWA data from the molecular genetics of schizophrenia (MGS) study. Two differentially expressed genes, *S100A8 *and *TYROBP*, reached nominal significance in gene-based association analysis.

## Materials and methods

### Subjects, sample preparation and sequencing

Schizophrenia patients were recruited from the pool of diagnosed schizophrenia patients of the inpatient unit of the VA Medical Center of Western New York (WNY) and from the Buffalo Psychiatric Center inpatient unit. All enrolled patients met the DSM-IV criteria for schizophrenia based on the examination of psychiatric case records and clinical interviews by at least two experienced psychiatrists. The healthy individuals were recruited though VA employees and advertisements in the local media. Individuals with a family history of schizophrenia or other major psychiatric disorders were excluded. A total of 26 subjects were included in this study. All subjects gave informed consent to participate in the study. The protocol and consent form were approved by the institutional review boards at the VA WNY Health Care System and New York State Office Mental Health.

Blood samples were collected with a 10 ml Vacutainer, Acid Citrate Dextrose (ACD) tube. Lymphocytes were prepared using isolymph (a diagnostic reagent used to separate lymphocytes from blood). Each 100 ml of isolymph contains 5.7 grams of Ficoll 400 and 9.0 grams of Diatrizoate Sodium) in a density gradient [20]. Ten ml of whole blood was diluted with equal volume RPMI and mixed by pipetting. Three milliliter of isolymph was transferred to a 15 ml Falcon conical bottom tube and 4 ml of diluted blood was carefully layered on top of the isolymph. The samples were centrifuged at 1640 rpm for 30 minutes. Lymphocytes were **c**ollected at the interface between the two layers by a sterile 1 ml pipette. The lymphocytes were mixed with 3 volumes of RPMI and centrifuged at 1400 rpm for 10 minutes. The supernatant was then removed. The lymphocytes were re-suspended in Cold Freeze Medium (60% RPMI 1640, 10% DMSO, 30% heat inactivated FCS) at a lymphocytes density of 6-12 × 10^6 ^cells/ml. The lymphocytes were distributed to NUNC plastic cryopreservation vials for long term storage. The vials were first placed in the cryo freezing container and stored at -80°C for at least 4 hours and then transferred to liquid nitrogen for final storage.

RNA was prepared from the frozen lymphocytes. Total RNA was extracted using the Mico-to-Midi Total RNA Purification System (Invitrogen, Carlsbad, CA) according to the manufacturer's instructions. The total RNA concentration and purity were determined spectrophotometrically at 260 nm and 280 nm in the Functional Genomics Shared Resource (FGSR) in Vanderbilt University.

MRNA capture, cDNA conversion, sizing, and library construction were performed using kits from the Illumina Company and by following the manufacture's recommended procedures. For RNA-Seq application, individual libraries were constructed for 3 schizophrenia patients, 3 controls, and 2 pools (10 subjects/pool) of schizophrenia patients and controls. Each library was loaded into a single lane of the Illumina Genome Analyzer II flow cell. For 3 cases and 3 controls, we performed paired-end sequencing while for pooled samples, we performed single-end sequencing. Image analysis and base-calling were performed by the Genome Analyzer Pipeline version 2.0 with default parameters [21]. Library construction and RNA sequencing was performed in the Genome Technology Core (GTC) in Vanderbilt University.

### Data process and analysis

After obtaining the short reads, we performed a series of quality checks, including quality score evaluation using program HTSeq [22] and marking duplicate reads by using software SAMTools [23]. All reads were independently aligned to a single reference file consisting of all human transcripts and the human genome in the UCSC genome assembly hg18 (NCBI build 36.1) by using TopHat (Version 1.0.10) [24]. The aligned sequences were evaluated with SAMTools for capture efficiency in order to ensure no artificial fragment representation (as assessed by fragment position distribution). We ran TopHat in the '*paired-end mode*' with the minimum distance between paired-end reads of 120 bp, a maximum distance of 500,000 bp, and default settings of other parameters (e.g., no more than two mismatches between read and reference were allowed in the first 28 bp (5' end) of the read).

To obtain an accurate measure of transcript abundance, we only used the reads that were uniquely mapped to the human genome. Since the sequence reads were paired-end, we quantified expression levels of all transcripts in each subject according to the fragments per kilobase of exon per million fragments mapped (FPKM), which was calculated by the software Cufflinks [25]. FPKM is a similar measurement to RPKM, which measures gene expression in Reads Per exon Kilobase per Million mapped reads (RPKM). RPKM has been used to normalize measurement of exon read density and allows transcript levels to be compared both within and between samples [25,26].

Considering that RNA-Seq mainly estimates exon expression and that most genes have multiple transcripts, it is necessary to determine how to estimate gene expression level based on transcript expression data. In our study, we employed a simple strategy: we first identified the differentially expressed transcripts (DETs) and then considered the unique genes of these transcripts as differentially expressed genes (DEGs) for further functional analysis.

To improve the reliability and comparability of differential expression analysis, we only examined the expression difference of those transcripts with FPKM value > 5 in all individually sequenced patients and controls [25]. Using these transcripts, we performed Fisher's exact test to identify transcripts with significantly differential expression between patients and controls [27-29]. For each transcript, we constructed a 2 × 2 contingency table, which included four FPKM values: *n, N-n, r, R-r *where *n *is the sum of FPKM values of a given transcript in 3 cases, *N *is the sum of FPKM values of all transcripts in cases, *r *is the sum of FPKM values of the given transcript in 3 controls, and *R *is the sum of the FPKM values of all transcripts in controls. To determine the expression change direction, we used "greater" or "less" parameters in the one-tailed Fisher's exact test to find the up-regulated transcripts or down-regulated transcripts respectively. Next, we controlled the type 1 errors by Bonferroni correction for the number of tests performed. A transcript was considered differentially expressed if the Bonferroni adjusted *P*-value was less than 0.05.

For data generated from pooled samples, we performed the same data processing and analysis as the individually sequenced samples except for using single-end mode to perform mapping of the reference sequence.

### Functional analysis

To assess the function of the DEGs that we identified, we conducted pathway enrichment tests for the DEGs using the online tool WebGestalt (version 2) [30]. We used all pathways in the Kyoto Encyclopedia of Genes and Genomes (KEGG) database. We selected those pathways having adjusted *P*-values of less than 0.01 calculated by the hypergeometric test followed by the Benjamini-Hochberg method [31], which was implemented in WebGestalt. To make the analysis biologically meaningful, we considered only those KEGG pathways containing 5 or more DEG genes.

To further systematically determine canonical signaling pathways and molecular networks that the DEGs might involve, we performed the pathway/network enrichment analysis using the Ingenuity Pathway Analysis (IPA) tool from the Ingenuity Systems [32]. For canonical signaling pathway analysis, given a list of genes, a right-tailed Fisher's exact test was performed for the enrichment of these genes in its hand-curated canonical pathway database. Here, the *P*-value calculated for a pathway measures the probability of being randomly selected from all of the curated pathways. To control the error rate in the analysis, IPA also provided a corrected *P*-values to identify the most significant results in IPA's canonical pathways based on the Benjamini-Hochberg method [31]. This tool allowed us to identify the signaling pathways in which the DEGs were enriched. In our study, we used a cut-off of the corrected *P*-value less than 0.05 (or score > 1.30, here score = -log *P*) to define the significant pathways. For network enrichment, the DEGs were overlaid onto a global molecular network (GMN) developed based on the Ingenuity Pathways Knowledge Base, in which functional relationships such as activation, chemical-protein interaction, expression, inhibition and regulation of binding were manually curated. Subnetworks of genes were then extracted from the GMN based on their connectivity using the algorithm developed by IPA [33]. For each subnetwork, a likelihood score, which measures the probability of the DEGs being found in the same subnetwork by chance, was transformed from the *P- *values calculated by Fisher's exact test. Additionally, the IPA assigned the top 3 biological functions for each network it identified.

### Gene-based genome-wide association analysis

The RNA-Seq application produced a list of differentially expressed genes in schizophrenia. We examined whether genetic variants in these DEGs harbored association signals. We conducted a gene-based GWA analysis using the MGS dataset for schizophrenia. We obtained this dataset from dbGaP under the protocol of "Genetic study of schizophrenia, nicotine dependence and other comorbid psychiatric disorders" by X.C. For each gene, its association *P*-value with schizophrenia was estimated using the VEGAS (Versatile Gene-based Association Study) software package [34].

## Results

### An overview of RNA-Seq data

In this study, we conducted genome-wide RNA sequencing for 6 individual samples and 2 pooled samples. The data from individually sequenced subjects was used for the initial identification of DEGs, while the pooled data was used to validate these DEGs.

For the 6 individually sequenced samples, after filtering by quality score, we generated an average of 8.7 million pairs of 43-bp paired-end reads per sample. The quality scores of the reads were satisfactory (Figure S1), of which 90.3**% **of the called bases had a Phred score ≥ 30. Table 1 shows the mapping statistics of the fragments. For each subject, an average of approximately 85.1% of the reads could be mapped to the human reference genome. Among the mapped sequences, ~48.6% of the read pairs were uniquely mapped to the human genome as properly aligned fragments. This is similar to the output of other RNA-Seq sequencing studies [35,36].

**Table 1:** Statistics of the number of fragments sequenced, aligned and mapped using TopHat

Sample	Sequenced fragments^a^	All mapped fragments (%)	Uniquely mapped fragments (%)^b^	Singleton fragments (%)^c^	Spliced fragments (%)^d^	Multi-loci mapped fragments (%)
Control						
1295-ZZ-4	10,114,082	84.5	45.2	24.9	3.8	26.1
1295-ZZ-9	6,152,569	82.0	46.1	24.5	3.3	26.2
1295-ZZ-13	9,263,358	86.5	50.6	25.0	3.2	21.2
Case						
1295-ZZ-21	9,287,780	85.8	50.7	25.1	3.3	20.9
1295-ZZ-32	8,911,007	85.4	48.5	26.3	4.0	21.2
1295-ZZ-36	8,211,577	86.6	50.7	23.3	3.2	22.8

^a^Each fragment has two short sequence reads.

^b^For uniquely mapped fragments, both of the two reads could be uniquely mapped to a unique location in the reference genome.

^c^For singleton fragments, only one of the two reads could be mapped to the reference genome.

^d^For spliced fragments, at least one of the two reads could be mapped across a splicing junction.

These reads were used to estimate transcript expression of all 6 samples. Table 2 shows the transcripts detected by RNA-Seq in subjects and mapped genes with FPKM values and coverage. Of the 33,599 transcripts and 32,797 genes annotated in the UCSC hg18, we detected 18,226 (54.2%) transcripts (FPKM > 0), which mapped to 14,929 (45.5%) unique genes. Among these transcripts, on average, 7223 (41.7%) had their FPKM values higher than 5.

**Table 2:** Statistics of the number of transcripts and genes detected

Sample	Number of transcripts	Number of genes	FPKM^a ^(mean ± sd)	Coverage^b ^(mean ± sd)	Number of genes with FPKM > 5
Control					
1295-ZZ-4	18,675	15,176	15.28 ± 85.28	8.54 ± 45.80	7876
1295-ZZ-9	17,134	14,330	14.36 ± 67.49	4.66 ± 21.03	6832
1295-ZZ-13	18,638	15,205	13.52 ± 68.07	7.06 ± 34.89	7507
Case					
1295-ZZ-21	18,274	14,883	14.86 ± 84.98	7.72 ± 43.89	6903
1295-ZZ-32	18,796	15,315	14.59 ± 167.18	7.14 ± 74.54	7359
1295-ZZ-36	17,837	14,668	11.84 ± 55.17	5.41 ± 24.17	6863
Pooled control	22,463	17,880	10.65 ± 70.63	18.04 ± 119.42	6134
Pooled case	22,256	17,620	11.67 ± 79.45	21.16 ± 143.79	6267

^a^For individually sequenced dataset, we used FPKM (Fragments Per Kilobase of exon per Million fragments mapped) to estimate transcript expression.

^b^The coverage was calculated based on the unit of transcript.

For the pooled samples, we generated a total of 26.2 million reads for the cases, of which 92.05% were mapped to the human reference genome, and 28.4 million reads for the controls, of which 91.29% were mapped. The resulting expression on transcription level and gene level were summarized in Table 2.

### Identification of differentially expressed genes

To identify the DEGs between the cases and controls, we used only those transcripts with FPKM values > 5.0 in all the 6 subjects. With this criterion, 4715 transcripts were included for differential expression analysis. Using Fisher's exact test, a total of 206 transcripts reached significance after Bonferroni correction (Table S1). Among these transcripts, 123 (mapped to 118 unique genes) were significantly down-regulated and 83 (mapped to 80 unique genes) were significantly up-regulated. In addition to transcripts expressed in both cases and controls, there were transcripts detected only in the cases or controls. Based on the FPKM distribution (Figure S2), transcripts exclusively expressed in either cases or controls with FPKM > 2.0 were included in the pathway and functional analyses. There were 12 transcripts exclusively expressed in cases (mapped to 12 unique genes) and 8 transcripts exclusively expressed in controls (mapped to 8 unique genes) (Table S2). Thus, we obtained a total of 218 genes differentially expressed among the 6 sequenced subjects.

### Validation and functional enrichment analysis of the DEGs

To validate the DEGs discovered from the individually sequenced dataset, we conducted similar differential expression analyses for the pooled dataset (see Materials and methods). There were 155 transcripts reaching nominal significance, of which 84 were up-regulated (mapped to 78 genes) and 72 were down-regulated (mapped to 68 unique genes). Of the 218 DEGs identified from the individually sequenced dataset, 9 were up-regulated (*GNAS, GNLY, HBA1, HBB, NCRNA00188, NEAT1, NFKB2, S100A8*, and *SNHG5*) and 10 were down-regulated genes (*CD74, CXCR4, LGALS2, LYZ, PF4, PIK3IP1, RBM38, RPL30, SCO2*, and *TYROBP*) that were found differentially expressed between cases and controls in the pooled dataset (with the same direction of gene expression change).

We conducted KEGG pathway profiling of these 218 DEGs. The results are shown in Table 3. Of the pathways enriched in these 218 genes, the most noticeable ones are involved in the immune and inflammation systems (antigen processing, cell adhesion molecules, hematopoietic cell lineage, systemic lupus erythematosus, chemokine signaling pathway, intestinal immune network for IgA production, toll-like receptor signaling pathway, T cell receptor signaling pathway, B cell receptor signaling pathway, Cytokine-cytokine receptor interaction, etc). Interestingly, the cell adhesion molecules pathway (CAMs, KEGG pathway ID hsa04514, adjusted *P *value = 5.78 × 10^-9^, Table 3) was the only pathway that was found to be significantly associated with both schizophrenia and bipolar disorder in a recent pathway analysis of schizophrenia and bipolar disorder GWAS datasets [37]. It was also highlighted in our recent pathway analysis using a generalized additive model for correction of gene length biases and other two methods (ALIGATOR and hypergeometric test) (unpublished data). Upon our further examination, we found 10 DEG genes in the CAMs pathway. Among the 10 genes, 8 were down-regulated (*CD4, HLA-DPA1, HLA-DRA, HLA-DRB1, ITGB2, PECAM1, SELL *and *VCAN*) and two were up-regulated (*CD8A *and *ITGB7*). Among the 8 down-regulated genes, three (*HLA-DPA1, HLA-DRA *and *HLA-DRB1*) were in the MHC region (chr6:20,000,000-40,000,000) (Table S3).

**Table 3:** KEGG pathways significantly enriched in the 218 differentially expressed genes

KEGG pathway	Number of genes (%)	Nominal *P*-value^a^	Adjusted *P*-value^b^
Antigen processing and presentation	14 (6.42)	9.27 × 10^-18^	1.58 × 10^-16^
Lysosome	11 (5.05)	1.07 × 10^-11^	9.09 × 10^-11^
Cell adhesion molecules (CAMs)	10 (4.59)	9.31 × 10^-10^	5.28 × 10^-9^
Hematopoietic cell lineage	8 (3.67)	9.68 × 10^-9^	4.11 × 10^-8^
MAPK signaling pathway	11 (5.05)	7.17 × 10^-8^	2.44 × 10^-7^
Chemokine signaling pathway	9 (4.13)	3.31 × 10^-7^	8.86 × 10^-7^
Systemic lupus erythematosus	8 (3.67)	3.65 × 10^-7^	8.86 × 10^-7^
Intestinal immune network for IgA production	5 (2.29)	3.89 × 10^-6^	8.27 × 10^-6^
Toll-like receptor signaling pathway	6 (2.75)	8.76 × 10^-6^	1.65 × 10^-5^
T cell receptor signaling pathway	6 (2.75)	1.29 × 10^-5^	2.19 × 10^-5^
Epithelial cell signaling in Helicobacter pylori infection	5 (2.29)	1.79 × 10^-5^	2.77 × 10^-5^
Leukocyte transendothelial migration	6 (2.75)	2.13 × 10^-5^	3.02 × 10^-5^
B cell receptor signaling pathway	5 (2.29)	2.88 × 10^-5^	3.77 × 10^-5^
Ribosome	5 (2.29)	6.23 × 10^-5^	7.56 × 10^-5^
Cytokine-cytokine receptor interaction	6 (2.75)	0.0017	0.0019
Metabolic pathways	13 (5.96)	0.0023	0.0024
Pathways in cancer	6 (2.75)	0.0049	0.0049

^a^Nominal *P*-value was calculated by hypergeometric test.

^b^Adjusted *P*-values was corrected of nominal *P*-values by Benjamini-Hochberg multiple testing correction.

**Figure 1:**
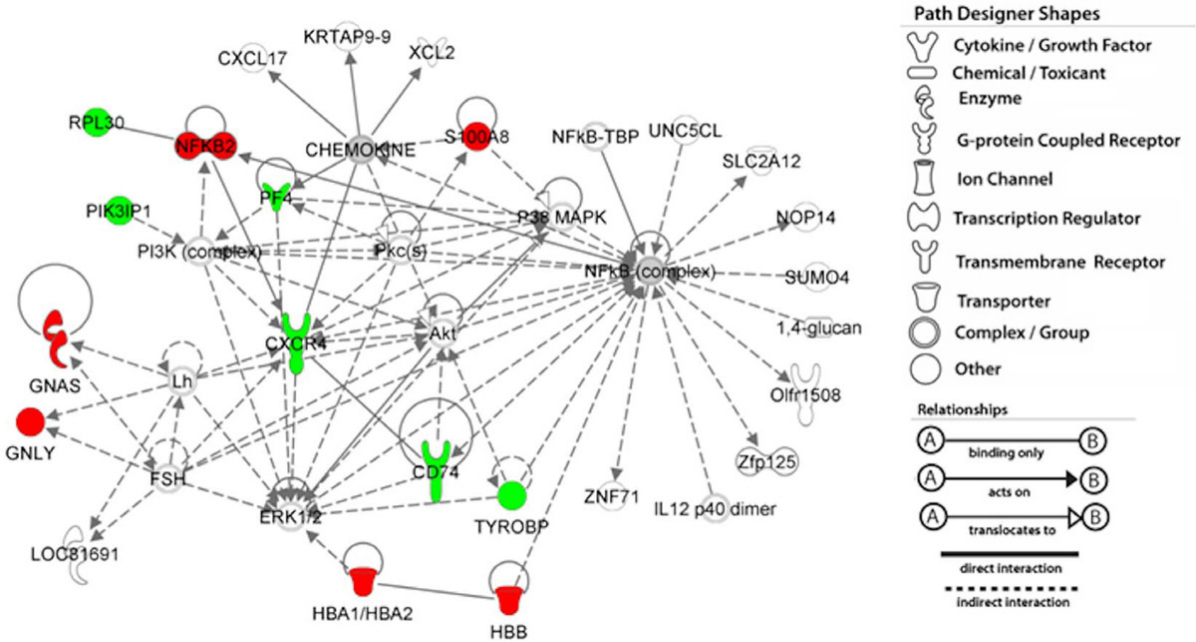
**The top network overrepresented by the 19 concordantly differentially expressed genes**. The functions of this network include "*molecular transport*," "*cellular movement*," and "*hematological system development and function*". Nodes in red indicate up-regulation in the cases and nodes in green indicate down-regulation.

For the 19 genes differentially expressed in both datasets, four are involved in immune systems (*CXCR4, NFKB2, PF4*, and *TYROBP*). In our KEGG pathway enrichment analysis, we found several pathways overrepresented in these genes including "Chemokine signaling pathway" and "Cytokine-cytokine receptor interaction," both of which were found significantly overrepresented in the 218 genes (Table 3). We further examined the pathways that were significantly overrepresented in these genes using IPA tools. The most significant pathways were "MIF-mediated glucocorticoid regulation," "MIF regulation of innate immunity," "TREM1 signaling," and "Induction of apoptosis by HIV1". MIF (macrophage migration inhibitory factor) is a unique counter-regulator of immunosuppressive and anti-inflammatory activities of glucocorticoids. Consistent with the results of 218 genes, all of these pathways are related to immune and inflammation systems. Furthermore, we conducted network analysis using IPA. Figure 1 shows the top network overrepresented in these genes, in which the top three functions are "Molecular transport," "Cellular movement," and "Hematological system development and function." Note that several genes in Figure 1 are potentially interesting like *CD74, S100A8, Akt, IL12, TYROBP, HBB *and *HBA1*. Among them, *CD74 *encodes a Type II transmembrane protein, which is a binding protein for MIF and an essential protein for MIF-induced activation of extracellular signal-regulated kinase-1/2MAP kinase cascade, cell proliferation and apoptosis [38].

### Gene-based association analyses of DEGs

One of the objectives of our RNA-Seq experiments was to test whether differentially expressed genes were enriched for association signals in GWA studies. Towards this goal, we conducted gene-based GWA analysis using the MGS dataset and the VEGAS method [34].This analysis produced a *P *value for each gene by considering gene's linkage disequilibrium information from the HapMap populations. We matched the 218 DEGs with those genes from the association analysis. Of the 218 genes, 21 had their *P *values less than 0.05 (Table 4). This was 2-fold enrichment than the expected (*P *= 0.0025). Five genes (*SELL *[39], *HLA-DRB1 *[40], *CEBPD *[41], *HSPA5 *[42], and *NRGN *[3]) from the matched list had been previously studied for schizophrenia with positive association signals, the rest of the genes were involved in immune responses or other neuronal diseases. Two genes, *S100A8 *and *TYROBP*, were differentially expressed in both the individual and pooled sequencing datasets.

**Table 4:** Association of differentially expressed genes with schizophrenia

Gene symbol	Association *P*-value^a^	Gene function
*LYN*	0.0025	Schizophrenia candidate
*HLA-DRB1*	0.0065	Schizophrenia candidate
*SAMHD1*	0.0112	Aicardi-Goutières syndrome
*SELL*	0.0114	Schizophrenia candidate
*S100A8*	0.0140	Immune response/disease
*CEBPD*	0.0179	Schizophrenia candidate
*ALDOA*	0.0215	Creutzfeldt-Jakob disease candidate
*CTSS*	0.0223	Immune response/disease
*NRGN*	0.0263	Schizophrenia candidate
*HLA-DRA*	0.0267	Schizophrenia candidate
*C1orf38*	0.0269	Breast cancer
*TRIM8*	0.0272	Ubiquitylation
*NKG7*	0.0283	Immune response/diseases
*MCL1*	0.0302	Immune response/disease
*FCN1*	0.0363	Rheumatoid arthritis candidate
*TYROBP*	0.0367	Immune response/diseases
*DDIT4*	0.0410	Parkinson's disease candidate
*FCGR3B*	0.0434	Immune response/disease
*LTA4H*	0.0454	Myocardial infarction candidate
*ITM2B*	0.0480	Alzheimer's disease candidate
*HSPA5*	0.0493	Schizophrenia candidate

^a^The association was examined using the MGS GWAS dataset (see Methods) by the VEGAS method.

## Discussion

Recent studies have shown that most genetic factors predisposing to schizophrenia have only a modest effect. GWA studies alone seem insufficient to identify the majority of these genetic factors. Expression level is an index of function of genes and may be useful for identifying risk genes for schizophrenia at the transciptomic level. In this study, we took advantage of recently available next generation sequencing technologies (i.e., RNA-Seq) to sequence poly-A tailed mRNAs from blood samples of 6 individuals and 2 pools of schizophrenia patients and controls. In the 6 individually sequenced samples, we found 218 genes showing differentially expression between cases and controls. Among these genes, 19 were nominally significant at the expression level in the 2 pooled samples. In our IPA analysis, we found that MIF regulation of innate immunity and TREM1 signaling were highly enriched in these 19 genes. Furthermore, of the 218 DEGs, 21 reached nominal significance in gene-based association analysis of the MGSGWAS dataset. Nineteen of these 21 genes are directly involved in immune response/diseases, or have been studied for candidates for schizophrenia and other neuronal diseases. Two genes, *S100A8 *and *TYROBP*, showed the same direction of expression changes in the individual and pooled sequencing datasets, and they also reached nominal significance in gene-based association analysis.

*S100A8*, also called *MRP-8*, encodes a calcium binding protein involved in inflammatory responses. It has been implicated in rheumatoid arthritis [43], systemic lupus erythematosus [44] and cancers [45,46]. Intriguingly, rheumatoid arthritis may be correlated with schizophrenia [47,48]. *TYROBP*, also known as *DAP12*, encodes an immunoreceptor adaptor protein that plays a key role in osteoclast differentiation and maturation [49,50]. Mutations in this gene lead to the Nasu-Hakola disease [51-54], a rare autosomal recessive disorder characterized by bone cyst and presenile dementia. In addition to their functions in the immune system, both genes are expressed in human brain. *S100A8 *shows elevated expression in cerebral ischemia [55] and posttraumatic brain injuries [56]. In a mouse model study, *S100A8 *expression increases significantly after chronic treatment with the antipsychotic drug olanzapine, which is used primarily to treat schizophrenia and bipolar disorder patients. *TYROBP *is implicated in the developmental neuronal death in hippocampus [57], impaired glutamatergic synaptic functions [58] and brain myelination [59]. All of these factors have been suspected to be involved in schizophrenia. *TYROBP *knockout mouse studies reveal deficits in cognitive functions and prepulse inhibition [49], symptoms that have been manifested in many schizophrenia patients. However, neither gene has been studied directly for schizophrenia. They may be novel candidates for the disease.

Glatt et al [60] applied microarray techniques to compare gene expression of peripheral blood cells (PBCs) and the dorsolateral prefrontal cortex (DLPFC) of the brain to identify risk factors for schizophrenia. They detected 123 differentially expressed genes in the blood samples. Among our 218 DEGs, 13 genes had the same direction of expression changes as reported by Glatt et al. Specifically, eight genes were down-regulated (*CD74, FCN1, FGR, HLA-DPA1, HLA-DRB1, IL10RA, PSAP*, and *ZFP36L2*) and five were up-regulated (*GOS2, HBA1, HBA2, HBB*, and *IL8*). The overlap of 13 genes with same direction of expression change is unlikely by chance considering they were selected from a genome-wide gene pool (*P*-value = 5.36 × 10^-6^). Interestingly, among the eight down-regulated genes, *CD74 *was consistently found down- regulated in three gene expression data sets (our individual sample, our pooled sample, and PBC sample in the Glatt et al study). Gene *CD74 *encodes a protein in MHC and is located in a region implicated by genome-wide linkage meta-analyses [61,62]. Additionally, the MHC locus on chromosome 6p was the most consistent finding from GWA studies [1-3]. Another gene, *HLA-DRB1*, also located in the MHC locus, was found to be differentially expressed in three data sets (the individual RNA-Seq dataset in this study, the PBC and DLPFC datasets in the Glatt et al study). *HLA-DRB1 *has been reported for positive association with schizophrenia [63,64]. We also found that SNPs influencing the expression of *HLA-DRB1 *(expression quantitative trait loci - eQTLs) were significantly associated with schizophrenia in the CATIE and MGS datasets (unpublished data). This result provides empirical evidence that a combination of GWA data and eQTL analysis may be effective to identify risk genes.

## Conclusion

This exploratory study aims at evaluating how RNA-Seq can be used to facilitate the identification of risk genes for complex diseases such as schizophrenia. Limitations include 1) the small number of subjects sequenced in this study and 2) only one pair of pooled samples was available to confirm the DEGs discovered in the individually sequenced dataset. Note that many RNA-Seq studies published in the past three years were based on a small number of samples (n ≤ 3) [65]. Due to these limitations, many genes potentially involved in schizophrenia could not be detected in the individually sequenced dataset and none of the genes in the pooled sample dataset reached significance after Bonferroni or false discovery rate correction. For these reasons, we selected to use those genes that reached nominal significance (one tailed test *P *< 0.1 for genes showing the same direction of expression changes) to verify the DEGs from the individually sequenced dataset. This may lead to the inclusion of some false positives in the 19 genes. At this time, we are unable to distinguish the true positives from the false ones. Since we observed 19 overlapping genes for the 218 DEGs, exceeding the expected number by chance, collectively, a majority of these 19 genes are unlikely to be false positives. The pathways and processes identified based on these 19 genes are likely reliable, and should provide important insights on the genes whose expression might be involved in the development of schizophrenia. Based on the same rationale, the list of genes identified by gene-based analysis may have false positives, but most of the genes could be considered promising candidates for schizophrenia. These promising candidates warrant further validation.

In summary, by combining high throughput RNA sequencing and GWA data, we have identified a list of candidate genes for schizophrenia despite our small sample size. These genes are enriched in the pathways and processes of the immune system. Our study demonstrates that integration of GWAS and gene expression can provide valuable information to prioritize candidates for future studies.

## Competing interests

The authors declare that they have no competing interests.

## Authors' contributions

JX, AL, MH SLD collected samples for the study. JS, JC, LW and SAB conducted data analysis. XC and ZZ conceived and designed the study and managed RNA-Seq experiments. JX, JS, ZZ and XC wrote the manuscript.

## Supplementary Material

Additional file 1 **This file includes the following figures and tables**. Figure S1 - Median Phred score vs. base position (cycle). Figure S2 - Distribution of the average FPKM values in the controls and cases. FPKM represents for fragments per kilobase of exon per million fragments mapped. Table S1 - Differentially expressed genes between schizophrenia patients and controls. Table S2 - Genes exclusively expressed in cases or controls. Table S3 - CAMs genes located in the MHC regions (chr6:20,000,000-40,000,000). Click here for file
