# Supplementary material for: RNA-Seq analysis implicates dysregulation of the immune system in schizophrenia
Source: BMC Genomics. 2012 Dec 17;13(Suppl 8):S2. doi: 10.1186/1471-2164-13-S8-S2 (PMC3535722; doi:10.1186/1471-2164-13-S8-S2)
Supplement: Additional file 1 — This file includes the following figures and tables. Figure S1 - Median Phred score vs. base position (cycle). Figure S2 - Distribution of the average FPKM values in the controls and cases. FPKM represents for fragments per kilobase of exon per million fragments mapped. Table S1 - Differentially expressed genes between schizophrenia patients and controls. Table S2 - Genes exclusively expressed in cases or controls. Table S3 - CAMs genes located in the MHC regions (chr6:20,000,000-40,000,000). [file 1471-2164-13-S8-S2-S1.pdf]

Additional file

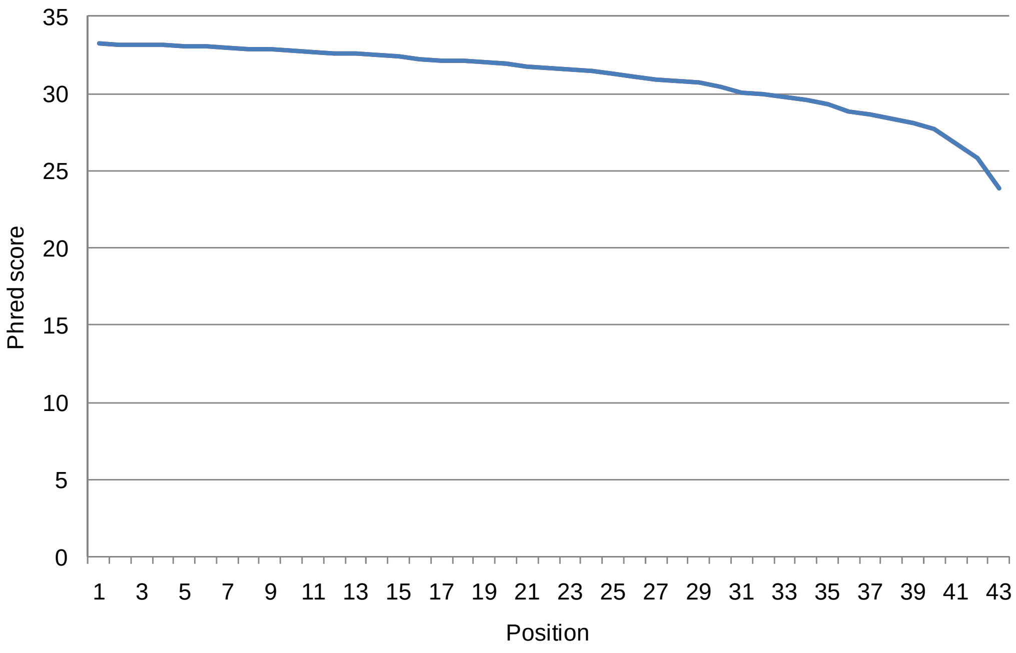

Figure S1. Median Phred score vs. base position (cycle)

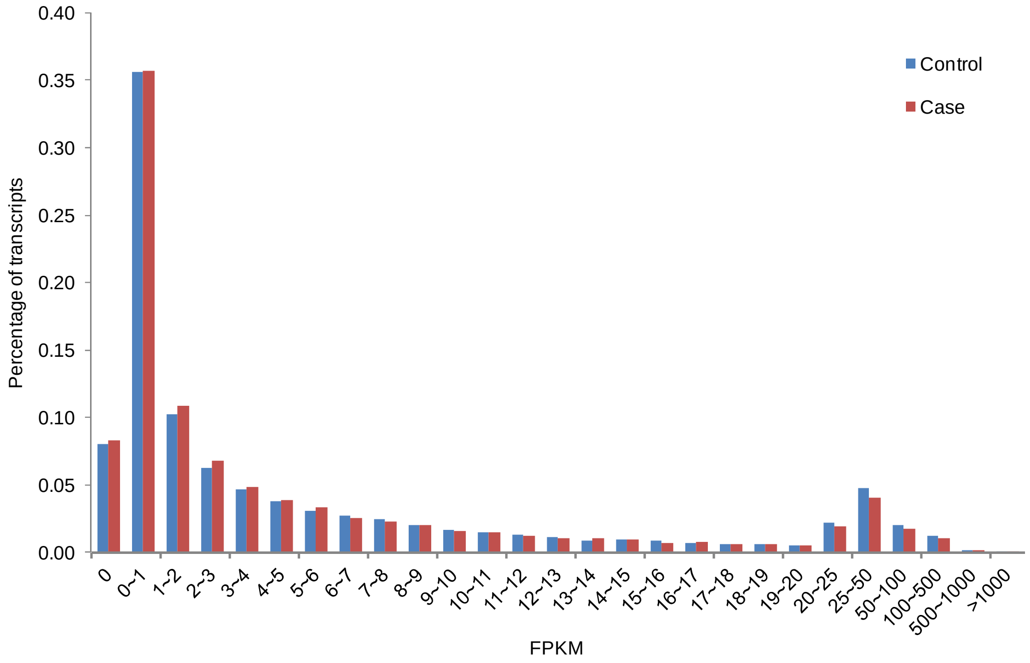

Figure S2. Distribution of the average FPKM values in the controls and cases. FPKM represents for fragments per kilobase of exon per million fragments mapped.

Table S1 - Differentially expressed genes between schizophrenia patients and controls

Transcript ID	Sum of control FPKM	Sum of case FPKM	*P*-value[a]	Bonferroni adjusted *P*-value	Gene symbol	Gene description
NM_000517	686.9	7778.5	0	0	*HBA2*	hemoglobin, alpha 2
NM_000518	835.4	11907.3	0	0	*HBB*	hemoglobin, beta
NM_000558	2304.3	15941.9	0	0	*HBA1*	hemoglobin, alpha 1
NM_006026	736.7	2742.9	5.53E-282	2.61E-278	*H1FX*	H1 histone family, member X
NM_005324	865.3	2765.4	3.94E-241	1.86E-237	*H3F3B*	H3 histone, family 3B (H3.3B)
NM_002228	213.0	1364.9	6.79E-213	3.20E-209	*JUN*	jun proto-oncogene
NM_005347	226.8	1346.8	1.06E-200	4.99E-197	*HSPA5*	heat shock 70kDa protein 5 (glucose-regulated protein, 78kDa)
NM_002778	4150.0	1985.0	1.70E-160	8.04E-157	*PSAP*	prosaposin
NM_000584	133.9	766.9	1.70E-112	8.01E-109	*IL8*	interleukin 8
NM_002003	3575.5	2014.0	5.37E-90	2.53E-86	*FCN1*	ficolin (collagen/fibrinogen domain containing)
NM_000239	7055.1	4773.2	1.09E-86	5.12E-83	*LYZ*	lysozyme
NM_005354	1546.4	2659.1	9.12E-74	4.30E-70	*JUND*	jun D proto-oncogene
NM_006472	3016.0	1760.5	6.30E-68	2.97E-64	*TXNIP*	thioredoxin interacting protein
NM_014330	377.1	979.3	8.15E-66	3.84E-62	*PPP1R15A*	protein phosphatase 1, regulatory (inhibitor) subunit 15A
NM_004083	110.9	472.4	2.37E-56	1.12E-52	*DDIT3*	DNA-damage-inducible transcript 3
NM_003407	497.7	1100.6	2.41E-56	1.13E-52	*ZFP36*	zinc finger protein 36, C3H type, homolog (mouse)
NM_006732	52.9	339.2	4.10E-54	1.93E-50	*FOSB*	FBJ murine osteosarcoma viral oncogene homolog B
NM_005319	31.9	285.2	1.15E-53	5.43E-50	*HIST1H1C*	histone cluster 1, H1c
NM_002970	892.9	1569.5	3.82E-47	1.80E-43	*SAT1*	spermidine/spermine N1-acetyltransferase 1
NM_004040	82.9	365.7	2.08E-45	9.82E-42	*RHOB*	ras homolog gene family, member B
NM_020529	828.7	1458.3	5.88E-44	2.77E-40	*NFKBIA*	nuclear factor of kappa light polypeptide gene enhancer in B-cells inhibitor, alpha
NM_002229	1652.8	2483.7	1.02E-43	4.80E-40	*JUNB*	jun B proto-oncogene
NM_001160125	90.2	347.1	1.83E-38	8.62E-35	*KLF6*	Kruppel-like factor 6
NM_004417	576.0	1066.8	2.30E-37	1.08E-33	*DUSP1*	dual specificity phosphatase 1
NM_004907	338.2	731.9	1.24E-36	5.84E-33	*IER2*	immediate early response 2
NM_001013699	151.6	439.5	2.04E-35	9.60E-32	*H3F3C*	H3 histone, family 3C
NM_021149	1796.4	1106.7	3.39E-34	1.60E-30	*COTL1*	coactosin-like 1 (Dictyostelium)
NM_019111	2931.9	2028.0	3.10E-33	1.46E-29	*HLA-DRA*	major histocompatibility complex, class II, DR alpha
NM_015675	253.1	581.4	1.01E-32	4.74E-29	*GADD45B*	growth arrest and DNA-damage-inducible, beta
NM_002985	285.0	621.0	1.03E-31	4.88E-28	*CCL5*	chemokine (C-C motif) ligand 5
NM_003517	59.6	242.6	1.22E-28	5.73E-25	*HIST2H2AC*	histone cluster 2, H2ac
NM_002087	594.1	266.3	5.00E-28	2.36E-24	*GRN*	granulin
NM_004079	858.6	449.8	5.58E-28	2.63E-24	*CTSS*	cathepsin S

NM_002964	4275.3	5181.1	1.02E-26	4.82E-23	*S100A8*	S100 calcium binding protein A8
NM_001300	235.3	502.5	3.62E-25	1.71E-21	*KLF6*	Kruppel-like factor 6
NM_001066	636.5	313.0	1.16E-24	5.45E-21	*TNFRSF1B*	tumor necrosis factor receptor superfamily, member 1B
NM_001174105	363.8	134.9	2.49E-24	1.18E-20	*CD14*	CD14 molecule
NM_021009	1236.6	1737.7	1.74E-23	8.19E-20	*UBC*	ubiquitin C
NM_001908	354.5	135.5	6.57E-23	3.10E-19	*CTSB*	cathepsin B
NM_000211	971.5	570.8	1.11E-22	5.22E-19	*ITGB2*	integrin, beta 2 (complement component 3 receptor 3 and 4 subunit)
NM_033423	50.1	193.3	3.84E-22	1.81E-18	*GZMH*	granzyme H (cathepsin G-like 2, protein h-CCPX)
NM_005318	70.4	229.0	4.16E-22	1.96E-18	*H1F0*	H1 histone family, member 0
NM_001145252	544.7	263.7	4.97E-22	2.34E-18	*CFP*	complement factor properdin
NM_001909	867.0	496.7	5.14E-22	2.42E-18	*CTSD*	cathepsin D
NM_001064	674.6	356.4	5.46E-22	2.57E-18	*TKT*	transketolase
NM_004355	2898.5	2175.4	1.30E-20	6.13E-17	*CD74*	CD74 molecule, major histocompatibility complex, class II invariant chain
NM_006332	1750.4	1209.5	1.36E-20	6.42E-17	*IFI30*	interferon, gamma-inducible protein 30
NM_006433	404.9	690.7	4.68E-20	2.21E-16	*GNLY*	granulysin
NR_028272	572.1	888.5	7.94E-19	3.74E-15	*NEAT1*	nuclear paraspeckle assembly transcript 1 (non-protein coding)
NM_006176	414.1	195.0	3.60E-18	1.70E-14	*NRGN*	neurogranin (protein kinase C substrate, RC3)
NM_022153	447.5	218.1	4.12E-18	1.94E-14	*C10ORF54*	chromosome 10 open reading frame 54
NR_002819	740.7	1079.7	8.63E-18	4.07E-14	*MALAT1*	metastasis associated lung adenocarcinoma transcript 1 (non-protein coding)
NM_001743	278.6	109.1	1.23E-17	5.82E-14	*CALM2*	calmodulin 2 (phosphorylase kinase, delta)
NM_001127491	712.9	416.0	1.83E-17	8.62E-14	*ITGB2*	integrin, beta 2 (complement component 3 receptor 3 and 4 subunit)
NM_006887	548.8	297.1	4.55E-17	2.15E-13	*ZFP36L2*	zinc finger protein 36, C3H type-like 2
NM_000099	1624.5	1150.7	4.60E-17	2.17E-13	*CST3*	cystatin C
NM_004049	131.3	295.5	6.81E-17	3.21E-13	*BCL2A1*	BCL2-related protein A1
NM_021127	36.8	142.0	1.28E-16	6.05E-13	*PMAIP1*	phorbol-12-myristate-13-acetate-induced protein 1
NM_001015881	599.8	897.1	1.32E-16	6.21E-13	*TSC22D3*	TSC22 domain family, member 3
NM_001105556	327.1	145.0	1.73E-16	8.16E-13	*C1ORF38*	chromosome 1 open reading frame 38
NM_031311	341.6	157.2	4.35E-16	2.05E-12	*CPVL*	carboxypeptidase, vitellogenic-like
NM_005248	294.5	125.5	4.65E-16	2.19E-12	*FGR*	Gardner-Rasheed feline sarcoma viral (v-fgr) oncogene homolog
NM_020307	408.7	657.1	4.82E-16	2.27E-12	*CCNL1*	cyclin L1
NM_000989	6505.1	5474.3	5.69E-16	2.68E-12	*RPL30*	ribosomal protein L30
NM_004233	73.7	201.6	5.99E-16	2.82E-12	*CD83*	CD83 molecule
NM_000442	267.1	109.2	7.96E-16	3.75E-12	*PECAM1*	platelet/endothelial cell adhesion molecule
NM_001164097	309.4	136.9	1.13E-15	5.31E-12	*VCAN*	versican

NM_001961	3332.4	2644.0	1.44E-15	6.77E-12	*EEF2*	eukaryotic translation elongation factor 2
NM_001126105	231.6	90.1	4.29E-15	2.02E-11	*SLC7A7*	solute carrier family 7 (cationic amino acid transporter, y+ system), member 7
NM_006762	1788.6	1317.4	4.86E-15	2.29E-11	*LAPTM5*	lysosomal protein transmembrane 5
NM_019058	555.9	321.2	1.97E-14	9.27E-11	*DDIT4*	DNA-damage-inducible transcript 4
NM_004343	523.4	776.0	3.40E-14	1.60E-10	*CALR*	calreticulin
NM_177924	308.4	146.4	9.14E-14	4.31E-10	*ASAH1*	N-acylsphingosine amidohydrolase (acid ceramidase) 1
NM_031419	149.9	298.9	1.17E-13	5.53E-10	*NFKBIZ*	nuclear factor of kappa light polypeptide gene enhancer in B-cells inhibitor, zeta
NM_001039396	324.0	158.3	1.38E-13	6.51E-10	*MPEG1*	macrophage expressed 1
NM_016127	932.3	624.1	1.51E-13	7.11E-10	*TMEM66*	transmembrane protein 66
NM_005195	301.1	142.7	1.98E-13	9.33E-10	*CEBPD*	CCAAT/enhancer binding protein (C/EBP), delta
NM_002339	980.0	665.1	2.30E-13	1.09E-09	*LSP1*	lymphocyte-specific protein 1
NM_001251	618.6	378.7	3.25E-13	1.53E-09	*CD68*	CD68 molecule
NM_002569	238.4	101.6	3.42E-13	1.61E-09	*FURIN*	furin (paired basic amino acid cleaving enzyme)
NM_005720	784.5	511.4	5.52E-13	2.60E-09	*ARPC1B*	actin related protein 2/3 complex, subunit 1B, 41kDa
NM_006498	343.7	176.1	6.67E-13	3.14E-09	*LGALS2*	lectin, galactoside-binding, soluble, 2
NM_033027	193.3	350.7	6.92E-13	3.26E-09	*CSRNP1*	cysteine-serine-rich nuclear protein 1
NM_001002235	181.7	67.7	7.66E-13	3.61E-09	*SERPINA1*	serpin peptidase inhibitor, clade A (alpha-1 antiproteinase, antitrypsin), member 1
NM_000999	1247.6	1587.5	8.03E-13	3.79E-09	*RPL38*	ribosomal protein L38
NM_000714	331.7	170.8	2.54E-12	1.20E-08	*TSPO*	translocator protein (18kDa)
NM_025195	23.0	94.3	4.29E-12	2.02E-08	*TRIB1*	tribbles homolog 1 (Drosophila)
NM_005346	74.9	177.6	9.69E-12	4.57E-08	*HSPA1B*	heat shock 70kDa protein 1B
NM_001173515	1879.2	1449.1	1.08E-11	5.07E-08	*TYROBP*	TYRO protein tyrosine kinase binding protein
NM_005601	347.9	533.7	1.52E-11	7.16E-08	*NKG7*	natural killer cell group 7 sequence
NM_015474	248.3	117.1	1.68E-11	7.92E-08	*SAMHD1*	SAM domain and HD domain 1
NM_000358	314.9	163.6	1.73E-11	8.17E-08	*TGFBI*	transforming growth factor, beta-induced, 68kDa
NM_005594	318.8	166.8	1.82E-11	8.58E-08	*NACA*	nascent polypeptide-associated complex alpha subunit
NM_000528	326.1	172.7	2.57E-11	1.21E-07	*MAN2B1*	mannosidase, alpha, class 2B, member 1
NM_006291	593.8	377.0	2.81E-11	1.33E-07	*TNFAIP2*	tumor necrosis factor, alpha-induced protein 2
NM_004108	235.0	109.6	3.80E-11	1.79E-07	*FCN2*	ficolin (collagen/fibrinogen domain containing lectin) 2 (hucolin)
NM_024829	281.1	142.3	3.95E-11	1.86E-07	*PLBD1*	phospholipase B domain containing 1
NM_001013	3344.9	2757.5	4.22E-11	1.99E-07	*RPS9*	ribosomal protein S9
NM_004235	85.2	187.8	5.12E-11	2.41E-07	*KLF4*	Kruppel-like factor 4 (gut)
NM_006931	155.4	284.4	6.45E-11	3.04E-07	*SLC2A3*	solute carrier family 2 (facilitated glucose transporter), member 3
NM_001025159	798.7	546.4	7.17E-11	3.38E-07	*CD74*	CD74 molecule, major histocompatibility complex, class II invariant chain

NM_001142277	205.6	91.6	7.64E-11	3.60E-07	*APLP2*	amyloid beta (A4) precursor-like protein 2
NM_005627	46.3	127.0	9.58E-11	4.52E-07	*SGK1*	serum/glucocorticoid regulated kinase 1
NM_000895	324.5	175.7	1.22E-10	5.77E-07	*LTA4H*	leukotriene A4 hydrolase
NM_000397	307.1	163.6	1.39E-10	6.58E-07	*CYBB*	cytochrome b-245, beta polypeptide
NM_003217	293.4	154.5	2.08E-10	9.82E-07	*TMBIM6*	transmembrane BAX inhibitor motif containing 6
NM_001781	225.4	367.6	3.20E-10	1.51E-06	*CD69*	CD69 molecule
NM_000101	1236.6	921.5	3.57E-10	1.68E-06	*CYBA*	cytochrome b-245, alpha polypeptide
NM_000690	210.7	99.1	4.02E-10	1.89E-06	*ALDH2*	aldehyde dehydrogenase 2 family (mitochondrial)
NM_002029	207.6	96.5	4.09E-10	1.93E-06	*FPR1*	formyl peptide receptor 1
NM_000616	343.1	193.5	4.54E-10	2.14E-06	*CD4*	CD4 molecule
NM_025092	599.3	392.6	4.68E-10	2.21E-06	*ATHL1*	ATH1, acid trehalase-like 1 (yeast)
NM_012483	56.7	138.5	1.02E-09	4.82E-06	*GNLY*	granulysin
NM_003516	28.6	93.2	1.09E-09	5.16E-06	*HIST2H2AA3*	histone cluster 2, H2aa3
NM_002032	737.2	510.7	1.57E-09	7.40E-06	*FTH1*	ferritin, heavy polypeptide 1
NM_002332	146.3	58.7	1.68E-09	7.94E-06	*LRP1*	low density lipoprotein receptor-related protein 1
NR_027856	46.9	120.8	1.89E-09	8.93E-06	*CLK1*	CDC-like kinase 1
NM_001130689	44.1	115.4	3.14E-09	1.48E-05	*HMGB2*	high-mobility group box 2
NM_007074	1178.1	889.5	4.45E-09	2.10E-05	*CORO1A*	coronin, actin binding protein, 1A
NM_002619	157.2	67.5	4.50E-09	2.12E-05	*PF4*	platelet factor 4
NM_002070	1060.7	791.4	6.03E-09	2.84E-05	*GNAI2*	guanine nucleotide binding protein (G protein), alpha inhibiting activity polypeptide 2
NM_002308	136.9	56.1	7.41E-09	3.49E-05	*LGALS9*	lectin, galactoside-binding, soluble, 9
NM_003254	343.2	204.2	9.23E-09	4.35E-05	*TIMP1*	TIMP metallopeptidase inhibitor 1
NM_001004431	171.1	78.5	9.87E-09	4.65E-05	*METRNL*	meteorin, glial cell differentiation regulator-like
NM_001336	856.2	621.9	1.27E-08	6.00E-05	*CTSZ*	cathepsin Z
NM_017495	158.6	72.2	1.63E-08	7.66E-05	*RBM38*	RNA binding motif protein 38
NM_014059	82.1	166.0	1.63E-08	7.70E-05	*C13ORF15*	chromosome 13 open reading frame 15
NM_024329	617.4	425.3	1.79E-08	8.42E-05	*EFHD2*	EF-hand domain family, member D2
NM_001142276	364.3	222.6	2.02E-08	9.55E-05	*APLP2*	amyloid beta (A4) precursor-like protein 2
NM_005252	628.7	818.3	2.47E-08	0.0001	*FOS*	FBJ murine osteosarcoma viral oncogene homolog
NM_001558	144.0	62.6	2.60E-08	0.0001	*IL10RA*	interleukin 10 receptor, alpha
NM_021999	759.6	546.3	2.76E-08	0.0001	*ITM2B*	integral membrane protein 2B
NM_004048	8808.0	9291.2	3.40E-08	0.0002	*B2M*	beta-2-microglobulin
NM_002124	1895.4	1540.7	7.13E-08	0.0003	*HLA-DRB1*	major histocompatibility complex, class II, DR beta 1
NM_015714	780.0	978.0	7.22E-08	0.0003	*G0S2*	G0/G1switch 2
NM_004313	350.0	216.8	7.34E-08	0.0003	*ARRB2*	arrestin, beta 2
NM_001928	253.2	143.1	7.54E-08	0.0004	*CFD*	complement factor D (adipsin)
NM_005475	148.1	68.5	7.83E-08	0.0004	*SH2B3*	SH2B adaptor protein 3
NM_054114	69.9	143.9	8.45E-08	0.0004	*TAGAP*	T-cell activation RhoGTPase activating protein
NM_001136135	1135.4	875.8	1.14E-07	0.0005	*RPL28*	ribosomal protein L28

NM_017740	213.9	116.4	1.37E-07	0.0006	*ZDHHC7*	zinc finger, DHHC-type containing 7
NM_018434	291.3	174.3	1.49E-07	0.0007	*RNF130*	ring finger protein 130
NM_001114620	114.2	47.3	1.60E-07	0.0008	*MGAT1*	mannosyl (alpha-1,3-)-glycoprotein beta-1,2-N-acetylglucosaminyltransferase
NM_006813	218.2	330.4	2.03E-07	0.0010	*PNRC1*	proline-rich nuclear receptor coactivator 1
NR_027855	184.1	287.5	2.26E-07	0.0011	*CLK1*	CDC-like kinase 1
NM_052880	378.2	245.6	3.99E-07	0.0019	*PIK3IP1*	phosphoinositide-3-kinase interacting protein 1
NM_001145373	44.5	100.6	5.69E-07	0.0027	*OTUD1*	OTU domain containing 1
NM_015481	188.7	101.6	6.02E-07	0.0028	*ZNF385A*	zinc finger protein 385A
NR_024151	116.0	50.9	6.31E-07	0.0030	*HSPA7*	heat shock 70kDa protein 7 (HSP70B)
NM_031950	46.7	104.8	6.52E-07	0.0031	*FGFBP2*	fibroblast growth factor binding protein 2
NM_001169109	195.3	107.0	7.42E-07	0.0035	*SCO2*	SCO cytochrome oxidase deficient homolog 2 (yeast)
NM_001114619	148.6	73.9	7.70E-07	0.0036	*MGAT1*	mannosyl (alpha-1,3-)-glycoprotein beta-1,2-N-acetylglucosaminyltransferase
NM_001623	873.1	666.8	1.20E-06	0.0056	*AIF1*	allograft inflammatory factor 1
NM_005729	98.4	171.5	1.40E-06	0.0066	*PPIF*	peptidylprolyl isomerase F
NM_004107	248.8	150.0	1.52E-06	0.0071	*FCGRT*	Fc fragment of IgG, receptor, transporter, alpha
NM_004635	155.4	79.7	1.53E-06	0.0072	*MAPKAPK3*	mitogen-activated protein kinase-activated protein kinase 3
NM_177401	270.4	380.5	1.59E-06	0.0075	*MIDN*	midnolin
NM_001024	820.7	995.8	1.66E-06	0.0078	*RPS21*	ribosomal protein S21
NM_184041	968.3	751.5	1.68E-06	0.0079	*ALDOA*	aldolase A, fructose-bisphosphate
NM_012401	227.3	133.5	1.93E-06	0.0091	*PLXNB2*	plexin B2
NR_003038	191.2	285.5	2.06E-06	0.0097	*SNHG5*	small nucleolar RNA host gene 5 (non-protein coding)
NM_001098526	294.6	186.9	2.06E-06	0.0097	*AMICA1*	adhesion molecule, interacts with CXADR antigen 1
NM_001077488	640.9	797.3	2.18E-06	0.0103	*GNAS*	GNAS complex locus
NM_001124	39.5	90.6	2.42E-06	0.0114	*ADM*	adrenomedullin
NM_005194	500.0	639.5	2.45E-06	0.0116	*CEBPB*	CCAAT/enhancer binding protein (C/EBP), beta
NM_000570	191.2	108.3	2.75E-06	0.0129	*FCGR3B*	Fc fragment of IgG, low affinity IIIb, receptor (CD16b)
NM_016270	484.0	620.9	2.86E-06	0.0135	*KLF2*	Kruppel-like factor 2 (lung)
NM_002654	279.9	177.1	3.23E-06	0.0152	*PKM2*	pyruvate kinase, muscle
NR_001298	218.0	128.8	3.32E-06	0.0157	*HLA-DRB6*	major histocompatibility complex, class II, DR beta 6 (pseudogene)
NM_014435	112.3	52.1	3.56E-06	0.0168	*NAAA*	N-acylethanolamine acid amidase
NM_017801	135.0	67.8	3.62E-06	0.0171	*CMTM6*	CKLF-like MARVEL transmembrane domain containing 6
NM_001136136	1264.1	1018.6	3.64E-06	0.0171	*RPL28*	ribosomal protein L28
NM_020801	72.1	133.7	3.93E-06	0.0185	*ARRDC3*	arrestin domain containing 3
NM_002727	1318.1	1069.0	4.49E-06	0.0212	*SRGN*	serglycin
NM_018837	166.5	91.2	4.55E-06	0.0214	*SULF2*	sulfatase 2

NM_033554	1093.6	870.8	4.66E-06	0.0220	*HLA-DPA1*	major histocompatibility complex, class II, DP alpha 1
NM_032048	141.1	72.9	4.80E-06	0.0227	*EMILIN2*	elastin microfibril interfacer 2
NM_025250	99.2	44.2	5.08E-06	0.0239	*TTYH3*	tweety homolog 3 (Drosophila)
NM_003467	884.0	688.4	5.30E-06	0.0250	*CXCR4*	chemokine (C-X-C motif) receptor 4
NM_152866	92.3	159.3	5.47E-06	0.0258	*MS4A1*	membrane-spanning 4-domains, subfamily A, member 1
NR_027168	191.4	280.7	5.54E-06	0.0261	*NCRNA00188*	non-protein coding RNA 188
NM_201413	103.1	47.1	5.90E-06	0.0278	*APP*	amyloid beta (A4) precursor protein
NR_027353	89.5	154.0	7.33E-06	0.0346	*CD8A*	CD8a molecule
NR_024110	61.5	118.5	8.28E-06	0.0391	*SBDSP1*	Homo sapiens Shwachman-Bodian-Diamond syndrome pseudogene 1
NM_001039465	48.8	100.1	8.78E-06	0.0414	*SRSF5*	serine/arginine-rich splicing factor 5
NM_145912	118.8	59.1	9.01E-06	0.0425	*NFAM1*	NFAT activating protein with ITAM motif 1
NM_002568	1692.0	1412.1	9.28E-06	0.0437	*PABPC1*	poly(A) binding protein, cytoplasmic 1
NM_004390	195.3	115.2	9.35E-06	0.0441	*CTSH*	cathepsin H
NM_145058	187.1	108.8	9.57E-06	0.0451	*RILPL2*	Rab interacting lysosomal protein-like 2
NM_003190	213.3	128.7	9.90E-06	0.0467	*TAPBP*	TAP binding protein (tapasin)
NM_212481	268.8	173.3	1.02E-05	0.0482	*ARID5A*	AT rich interactive domain 5A (MRF1-like)
NM_003641	493.1	620.5	1.07E-05	0.0502	*IFITM1*	interferon induced transmembrane protein 1 (9-27)
NM_178841	123.8	62.6	1.09E-05	0.0513	*RNF166*	ring finger protein 166
NM_013262	59.8	114.2	1.21E-05	0.0573	*MYLIP*	myosin regulatory light chain interacting protein
NM_001111097	208.2	126.3	1.256E-05	0.0592	*LYN*	v-yes-1 Yamaguchi sarcoma viral related oncogene homolog
NM_000732	232.8	326.2	1.27E-05	0.0597	*CD3D*	CD3d molecule, delta (CD3-TCR complex)
NM_002076	96.3	43.9	1.31E-05	0.0620	*GNS*	glucosamine (N-acetyl)-6-sulfatase
NM_001031696	108.9	52.5	1.36E-05	0.0640	*PLD3*	phospholipase D family, member 3
NM_000889	99.2	164.2	1.47E-05	0.0691	*ITGB7*	integrin, beta 7
NM_182763	133.4	206.2	1.54E-05	0.0727	*MCL1*	myeloid cell leukemia sequence 1 (BCL2-related)
NM_002957	94.0	42.7	1.55E-05	0.0730	*RXRA*	retinoid X receptor, alpha
NM_030912	131.0	68.8	1.58E-05	0.0746	*TRIM8*	tripartite motif-containing 8
NM_001134486	33.2	75.1	1.83E-05	0.0863	*GBP5*	guanylate binding protein 5
NM_181672	236.0	326.7	1.94E-05	0.0916	*OGT*	O-linked N-acetylglucosamine (GlcNAc) transferase (UDP-N-acetylglucosamine:polypeptide-N-acetylglucosaminyl transferase)
NM_003380	1662.0	1394.2	1.99E-05	0.0938	*VIM*	vimentin
NR_029467	259.5	168.2	2.07E-05	0.0974	*SELL*	selectin L

[a]*P*-value was calculated by Fisher's exact test.

Table S2 - Genes exclusively expressed in cases or controls

ID	Control 1	Control 2	Control 2	Case 1	Case 2	Case 3	Gene	Description
NM_001077494	0.0000	0.0000	0.0000	11.6300	7.7593	10.4268	*NFKB2*	nuclear factor of kappa light polypeptide gene enhancer in B-cells 2 (p49/p100)
NM_001127213	0.0000	0.0000	0.0000	106.5230	46.4870	40.8874	*LY6E*	lymphocyte antigen 6 complex, locus E
NM_001128208	0.0000	0.0000	0.0000	6.0587	2.5580	5.8632	*LEPROTL1*	leptin receptor overlapping transcript-like 1
NM_001144876	0.0000	0.0000	0.0000	2.0537	2.8688	9.1984	*DOK3*	docking protein 3
NM_001146068	0.0000	0.0000	0.0000	12.3968	22.5249	3.4852	*CAPN2*	calpain 2, (m/II) large subunit
NM_001921	0.0000	0.0000	0.0000	11.7537	6.8281	7.4528	*DCTD*	dCMP deaminase
NM_006117	0.0000	0.0000	0.0000	4.0215	4.7377	7.7940	*PECI*	peroxisomal D3,D2-enoyl-CoA isomerase
NM_138473	0.0000	0.0000	0.0000	5.5412	9.4535	7.2871	*SP1*	Sp1 transcription factor
NM_182835	0.0000	0.0000	0.0000	5.0806	2.3511	4.5758	*SCFD1*	sec1 family domain containing 1
NM_194450	0.0000	0.0000	0.0000	11.6918	8.0243	2.5439	*CLEC4A*	C-type lectin domain family 4, member A
NM_198218	0.0000	0.0000	0.0000	4.6926	2.6236	2.1580	*ING1*	inhibitor of growth family, member 1
NM_207002	0.0000	0.0000	0.0000	5.6596	2.5886	4.3098	*BCL2L11*	BCL2-like 11 (apoptosis facilitator)
NM_001001390	11.6376	5.4548	10.8171	0.0000	0.0000	0.0000	*CD44*	CD44 molecule (Indian blood group)
NM_001145134	3.3259	6.4170	3.3070	0.0000	0.0000	0.0000	*CPT1B*	carnitine palmitoyltransferase 1B (muscle)
NM_001146110	3.2340	3.7043	3.9840	0.0000	0.0000	0.0000	*MIER1*	mesoderm induction early response 1 homolog (Xenopus laevis)
NM_005133	10.2785	5.2491	8.6697	0.0000	0.0000	0.0000	*RCE1*	RCE1 homolog, prenyl protein peptidase (S. cerevisiae)
NM_152236	7.9148	6.8971	5.1274	0.0000	0.0000	0.0000	*GAS2L1*	growth arrest-specific 2 like 1
NR_024040	2.2370	4.1330	4.8507	0.0000	0.0000	0.0000	*CSGALNACT1*	chondroitin sulfate N-acetylgalactosaminyltransferase 1
NR_027299	2.2421	11.6814	2.9371	0.0000	0.0000	0.0000	*XPC*	xeroderma pigmentosum, complementation group C
NR_027834	8.3139	8.9451	5.4076	0.0000	0.0000	0.0000	*APOL3*	apolipoprotein L, 3

Table S3 - CAMs genes located in the MHC regions (chr6:20,000,000-40,000,000)

Gene	Start position	End position
HLA-F	29799096	29803052
HLA-G	29903497	29906859
HLA-A	30018310	30021633
HLA-E	30565250	30569077
HLA-C	31344508	31347834
HLA-B	31429628	31432914
HLA-DRA	32515625	32520801
HLA-DRB5	32593129	32605984
HLA-DRB1	32654527	32665559
HLA-DQA1	32713161	32719407
HLA-DQB1	32735635	32742444
HLA-DQA2	32817141	32823199
HLA-DOB	32888518	32892803
HLA-DMB	33010393	33016795
HLA-DMA	33024373	33028831
HLA-DOA	33079937	33085367
HLA-DPA1	33140772	33149356
HLA-DPB1	33151738	33162954
